# The SLICC/ACR damage index, assessed using a community-based electronic healthcare record, predicts mortality in SLE patients: results from the United Kingdom Clinical Practice Research Datalink

**DOI:** 10.1093/rap/rkag079

**Published:** 2026-07-13

**Authors:** Jessica Ellis, Neil McHugh, John D Pauling, Ian N Bruce, Shivani Gor, Jenny H Humphreys, Hannah Vaughan-Williams, Ellie Korendowych, Christine MacFadyen, Sarah Skeoch, Anita McGrogan

**Affiliations:** Department of Life Sciences, University of Bath, Bath, UK; Department of Rheumatology, Royal National Hospital for Rheumatic Diseases, Royal United Hospitals Bath NHS Foundation Trust, Bath, UK; Department of Life Sciences, University of Bath, Bath, UK; Department of Life Sciences, University of Bath, Bath, UK; Department of Rheumatology, North Bristol NHS Trust, Bristol, UK; Centre for Musculoskeletal Research, Division of Musculoskeletal and Dermatological Sciences, University of Manchester, Manchester Academic Health Science Centre, Manchester, UK; Centre for Public Health, Faculty of Medicine, Health and Life Sciences, Queen’s University Belfast, Belfast, UK; Department of Rheumatology, Royal National Hospital for Rheumatic Diseases, Royal United Hospitals Bath NHS Foundation Trust, Bath, UK; Centre for Musculoskeletal Research, Division of Musculoskeletal and Dermatological Sciences, University of Manchester, Manchester Academic Health Science Centre, Manchester, UK; Department of Rheumatology, Royal National Hospital for Rheumatic Diseases, Royal United Hospitals Bath NHS Foundation Trust, Bath, UK; Department of Life Sciences, University of Bath, Bath, UK; Department of Rheumatology, Royal National Hospital for Rheumatic Diseases, Royal United Hospitals Bath NHS Foundation Trust, Bath, UK; Department of Life Sciences, University of Bath, Bath, UK; Department of Rheumatology, Royal National Hospital for Rheumatic Diseases, Royal United Hospitals Bath NHS Foundation Trust, Bath, UK; Department of Life Sciences, University of Bath, Bath, UK; Department of Rheumatology, Royal National Hospital for Rheumatic Diseases, Royal United Hospitals Bath NHS Foundation Trust, Bath, UK; Department of Life Sciences, University of Bath, Bath, UK

**Keywords:** systemic lupus erythematosus, United Kingdom, damage, mortality, epidemiology, observational study

## Abstract

**Objectives:**

SLE, the exemplar autoimmune multisystem disease, is still burdened by excess morbidity and mortality. Damage is a key mediator of this. Studies of damage in outside-of-hospital cohorts are lacking, limiting understanding of real-world patient outcomes. The objectives were thus to design an instrument for measurement of SLE organ damage (eSDI) usable within a UK electronic primary care healthcare database [Clinical Practice Research Datalink (CPRD)] and describe the accrual of damage and mortality associations in an SLE cohort.

**Methods:**

A cohort of SLE individuals, including incident and prevalent cases, was identified in the CPRD. Organ damage item definitions were made usable for the electronic registry setting. Prevalence of damage in the overall cohort was described using descriptive statistics. Associations with mortality and damage (expressed in two ways, SDI > 0 ever, or modelled as a continuous cumulative variable) were examined using multivariable logistic regression, Kaplan–Meier analysis and extended Cox proportional hazards analysis in the incident SLE population.

**Results:**

We identified 8363 SLE patients in total, of whom the eSDI measured damage in 3537 (42.3%). The most common items were osteoporosis (10.39%), malignancy (8.4%) and cataract (7.4%). Damage across all organ systems was associated with an increased odds of death (e.g. malignancy [odds ratio 6.33 (95% CI 5.34, 7.49)]. Development of damage (eSDI >0 or as a numerical damage score) was significantly associated with an increased hazard of mortality.

**Conclusion:**

The use of an adapted eSDI for community electronic health records is feasible and enables widening of the study of damage and its morbidity and mortality consequences to primary care populations. This is more likely to reflect the real-world accumulation and outcomes of damage over time in SLE.

Key messages

- Measurement of damage in SLE is feasible using UK routinely collected healthcare data and a modified SLICC-ACR Damage Index (eSDI).
- Musculoskeletal, ocular and cardiovascular domains were the most frequently affected, albeit at a lower prevalence than reported from specialist lupus cohorts.
- In a primary care setting, SLE-related organ damage is strongly associated with mortality.

## Introduction

SLE is a complex autoimmune disease with numerous manifestations. Improved understanding of pathophysiology and a broadening range of treatments have extended the life expectancy of most patients [1]. Unfortunately, SLE still carries significant morbidity. Damage, the irreversible loss of organ function irrespective of cause, subsequent to SLE diagnosis is a key determinant of morbidity [2]. The SLICC-ACR Damage Index (SDI) is the only validated clinician-completed tool to measure damage [3] providing a comprehensive assessment of important sequelae arising after SLE diagnosis [4]. Damage continues to accrue over time and is associated with numerous adverse consequences, including mortality [5]. Much of the evidence about damage is from hospital-based research cohorts. While these benefit from rigorous, systematic data collection, they tend to be biased towards moderate–severe SLE. The high economic costs of maintaining these cohorts can limit their size and the diversity of patients involved. Additionally, bespoke cohorts are at risk of selection bias; engaging in formal research programs is resource intensive and poor representativeness of underserved populations can be a problem. In contrast, most individuals in the UK are registered with primary care across their lifespan. Routinely collected data from these interactions are available for research use. These datasets provide a source of cost-effective longitudinal data, offering access to large numbers of unselected patients, potentially more indicative of the overall SLE population.

We aimed to adapt the SDI for use in a primary care electronic healthcare dataset (eSDI), then to use this to describe damage and its associations with mortality in a large UK population.

## Methods

### Study setting

This was a retrospective observational study performed within a large UK primary care research dataset. The study period was 1 January 1990 to 31 December 2020. Routinely collected data, recorded by UK general practitioners as part of usual patient care, is provided by the Clinical Practice Research Datalink (CPRD) in an anonymised form for research. CPRD GOLD, used in this study, provides information including diagnoses, prescriptions and test results, with 3.2 million active patients in 2021 [6] and a median follow-up time of 13 years [7].

### Ethical approval

Individual patient consent is not required for using the CPRD. The study was approved under the CPRD Research Data Governance process (protocol 21_000697) with approval given on 2 March 2022.

### Study participants

Participants were eligible if they were permanently registered adults (age ≥18 years at study entry) with at least 12 months of continuous data within the study period of a standard suitable for research. SLE participants were categorised using our published algorithm [8]. The index date was set as the earliest record of an SLE diagnostic code or code for immunosuppressant prescription. Study entry was set as the index date. Study exit was set as the earliest of death or the last data collection point (end of data collection or date of leaving the CPRD) or the end of the study period. Participants were incident if they had ≥12 months of CPRD data of a standard suitable for research preceding the index date containing no SLE diagnostic code, in keeping with other studies of incident SLE populations within the CPRD [9, 10]. The remaining patients were categorised as prevalent. Individuals with a calculated age at index >80 years were categorised as prevalent and deemed to pose a risk of inaccurately recorded index dates. Supplementary Fig. S1, Supplementary File S1 shows the study design.

### Damage assessment

SDI item definitions were transformed to make them usable within the CPRD (eSDI). Published phenotyping algorithms informed definitions where available [11]. Definitions were constructed using combinations of diagnostic, procedural, test and medication codes. Definitions were agreed upon by a team encompassing multiple disciplines including rheumatology, general practice and epidemiology with experience in SLE. Item scoring was completed as per the SDI. Items were not scored as damage if occurring prior to the index date. The presence of permanent conditions, e.g. diabetes, prior to the index precluded scoring of a post-index record as damage. Code lists for items are provided in Supplementary File S2.

### Covariates

Smoking status at the index date was categorised as never or ever using an in-house algorithm. BMI was the measurement recorded nearest to the index date, within a ±2-year window. Records were reviewed for available ethnicity Read codes and categorised for analyses as ‘White’ or ‘All other ethnic groups combined’ (due to small counts in non-aggregated ethnic minority categories). Missing data were handled using multivariate imputation by chained equations. Age at index (proxy for diagnosis) and sex (assigned at birth as recorded in CPRD) were covariates, as these have been shown to influence both damage and mortality [12]. Damage items coded before the index date deemed associated with mortality were considered comorbidities and included as baseline covariates.

### Statistical analysis

Descriptive statistics were used to summarise study population characteristics. As per CPRD guidance, counts <5 are not reported. Recording of damage items are reported as prevalence per 100 (with 95% CIs) for the total population. Continuous variables were compared using Welch’s two-sample *t*-test. Categorical variables were compared by the chi-squared test. Subsequent analyses were conducted in the incident population only.

Damage trends (average cohort total and organ domain damage) and mortality associations were assessed. Associations between individual items, organ domain involvement and any damage (SDI ≥ 0) with mortality (binary categorical, death ever) were initially explored using univariate logistic regression. Results were presented as odds ratios (ORs) with 95% CIs. Least absolute shrinkage and selection operator (LASSO) regression, including demographic variables and all damage items recorded, both pre- (i.e. comorbidities) and post-index, was conducted to identify other important variables to include, allowing appropriate shrinkage of coefficients towards zero to prevent overfitting due to large numbers of variables. Extra variables were added to the model and tested for multicollinearity and those significantly improving model fit (reduction in AIC >2) were retained. Multivariable regression models were constructed using covariates specified a priori (i.e. demographic and clinical comorbidities deemed likely to contribute to damage and mortality).

Cumulative probability of death-free survival from the index date was examined using Kaplan–Meier analysis. For those with no damage or death, data were censored at study exit. Comparison of survival between groups was made by the logrank test. Cox proportional hazards regression analysis was used to assess the relationship between damage and survival from the index date. Damage was incorporated as a time-varying covariate, with two damage states modelled: binary (SDI > 0 *vs* SDI = 0) and continuous cumulative variables. Age at index, sex, ethnicity, smoking and comorbidities at baseline were covariates in both. The proportional hazards assumption was tested using Schoenfeld residuals with stratification by sex and splitting of follow-up time used to produce the best models. Results are presented as hazard ratios (HRs) with 95% CIs. Analyses were conducted using R version 4.4.2 (R Foundation for Statistical Computing, Vienna, Austria) (Supplementary Table S1, Supplementary File S1 for package details).

## Results

There were 8363 patients in the study population. The cohort included 7320 women (87.5%) and the mean age at index was 46.7 years (s.d. 16.0; median 45.6). The median time in the study was 110.5 months (interquartile range 57.2–177.2). Characteristics of the study population are presented in Table 1. There was a substantial amount of missing data for BMI and ethnicity. Smoking data were missing in 3.3% of patients. Of the total cohort, 5097 (60.9%) were incident patients. During the study, 1004 (12%) patients died.

**Table 1:** Study population characteristics.

Characteristics		Values
Patients, *N*		8363
Sex, *n* (%)	Female	7320 (87.5)
	Male	1043 (12.5)
Age at index, years, mean (s.d.)		46.7 (16.0)
Ethnicity, *n* (%)	White	3502 (41.9)
	Black	195 (2.3)
	South Asian	271 (3.2)
	Mixed ethnic groups	39 (0.5)
	Other ethnic group	58 (0.7)
	Missing	4298 (51.4)
Smoking, *n* (%)	Never smoker	4096 (49.0)
	Ever smoker	3989 (47.7)
	Missing	278 (3.3)
BMI	Mean (s.d.)	26.9 (6.3)
	Missing, *n* (%)	3502 (41.9)
Time in study, weeks, mean (s.d.)		539.2 (349.2)
Patient status, *n* (%)	Alive	7359 (88.0)
	Deceased	1004 (12.0)
Age at death, years, mean (s.d.)		71.2 (13.8)
Medications (ever), *n* (%)	Glucocorticoid	5230 (62.5)
	Hydroxychloroquine	5738 (68.6)
	Chloroquine	117 (1.4)
	Azathioprine	1550 (18.5)
	Methotrexate	1159 (13.9)
	Leflunomide	125(1.5)
	Mycophenolate	754 (9)
	Ciclosporin	112 (1.3)
	Tacrolimus	75 (0.9)
	Cyclophosphamide	73 (0.9)
	Rituximab	16 (0.2)
	Belimumab	<5

Definitions for items in the eSDI are provided in Supplementary Table S2, Supplementary File S1. It was not possible to create a usable definition for shrinking lung syndrome. Three other items (pleural fibrosis, pulmonary infarct, chronic peritonitis) were not observed in any record. Four items [mesenteric insufficiency, avascular necrosis (second event), extensive scarring of the panniculum and premature gonadal failure] were each recorded in fewer than five participants.

Over the follow-up, 42.3% of SLE participants developed damage (eSDI >0). The range of maximum eSDI scores accrued over the follow-up are shown in Table 2. Of those with damage, the most common result was eSDI = 1 (range 1–9).

**Table 2:** Highest eSDI score during study follow-up.

Maximum eSDI score	*n* (%)
0	4826 (57.7)
1	1807 (21.6)
2	864 (10.3)
3	424 (5.1)
4	232 (2.8)
5	105 (1.3)
6	58 (0.7)
7	20 (0.2)
8	22 (0.3)
9	5 (0.1)

The prevalence (per 100 persons) of individual damage items is shown in Table 3. The most common items were osteoporosis [*n* = 869 (10.39%)], malignancy [*n* = 701 (8.4%)] and cataract [*n* = 617 (7.4%)]. Twenty-six individual items had a frequency of <1%.

**Table 3:** Prevalence of SDI items and univariate logistic regression with mortality.

Characteristics	*n*	Prevalence per 100 cases (95% CI)	OR (95% CI), *P*-value
Ocular			
Any cataract ever	617	7.38 (6.83, 7.96)	3.09 (2.55, 3.74), <0.001
Retinal change or optic atrophy	333	3.98 (3.57, 4.42)	2.61 (2.01, 3.36), <0.001
Neuropsychiatric			
Cognitive impairment	161	1.93 (1.64, 2.24)	3.63 (2.58, 5.06), <0.001
Seizures requiring therapy for 6 months	95	1.14 (0.92, 1.39)	1.73 (1.00, 2.83), 0.039
Cerebrovascular accident ever	281	3.36 (2.98, 3.77)	3.43 (2.57, 4.54), <0.001
Cerebrovascular accident ever (second event)	51	0.61 (0.45, 0.80)	3.91 (2.04, 7.17), <0.001
Cranial or peripheral neuropathy (excluding optic)	148	1.77 (1.50, 2.08)	1.89 (1.24, 2.80), 0.002
Transverse myelitis	8	0.10 (0.04, 0.19)	2.45 (0.36, 10.63), 0.274
Renal^a^			
Estimated or measured GFR <50%	463	5.54 (5.06, 6.05)	4.73 (3.86, 5.79), <0.001^b^
Proteinuria ≥3.5 g/24 h	108	1.29 (1.06, 1.56)	1.87 (0.43, 5.74) 0.327^c^
End-stage renal disease (regardless of dialysis or transplantation)	103	1.23 (1.01, 1.49)	4.68 (3.07, 7.02), <0.001^d^
Pulmonary			
Pulmonary hypertension	38	0.45 (0.32, 0.62)	3.01 (1.43, 5.92), 0.002
Pulmonary fibrosis	89	1.06 (0.86, 1.31)	4.89 (3.15, 7.48), <0.001
Shrinking lung	0	0.00	–^e^
Pleural fibrosis	0	0.00	–^e^
Pulmonary infarction	0	0.00	–^e^
Cardiovascular			
Angina or coronary artery bypass	257	3.07 (2.71, 3.47)	3.06 (2.30, 4.03), <0.001
Myocardial infarction ever	275	3.29 (2.92, 3.69)	4.64 (3.53, 6.08), <0.001
Myocardial infarction ever (second event)	36	0.43 (0.30, 0.60)	5.07 (2.53, 9.84), <0.001
Cardiomyopathy	296	3.54 (3.15, 3.96)	6.95 (5.46, 8.82), <0.001
Valvular disease	235	2.81 (2.47, 3.19)	3.94 (2.97, 5.20), <0.001
Pericarditis for 6 months or pericardiectomy	8	0.10 (0.04, 0.19)	–^e^
Peripheral vascular			
Claudication for 6 months	139	1.66 (1.40, 1.96)	3.75 (2.60, 5.34), <0.001
Minor tissue loss (pulp space)	7	0.08 (0.03, 0.17)	9.81 (2.16, 49.86), 0.003
Significant tissue loss ever	31	0.37 (0.25, 0.53)	6.82 (2.95, 15.60), <0.001
Significant tissue loss ever (second event)	8	0.10 (0.04, 0.19)	22.33 (5.14, 152.52), <0.001
Venous thrombosis with swelling, ulceration or venous stasis	223	2.67 (2.33, 3.04)	2.87 (2.11, 3.87), <0.001
Gastrointestinal			
Infarction or resection of bowel below duodenum, spleen, liver or gall bladder ever for any cause	344	4.11 (3.70, 4.56)	1.76 (1.32, 2.32), <0.001
Infarction or resection of bowel below duodenum, spleen, liver or gall bladder ever for any cause (second event)	6	0.07 (0.03, 0.16)	1.51 (0.08, 9.36), 0.708
Mesenteric insufficiency	<5	–	7.34 (0.29, 185.61), 0.159
Chronic peritonitis	0	0.00	1.83 (0.28, 7.33), 0.443
Stricture or upper gastrointestinal tract surgery ever	72	0.86 (0.67, 1.08)	2.47 (1.40, 4.14), 0.001
Chronic pancreatitis	10	0.12 (0.06, 0.22)	1.83 (0.28, 7.33), 0.443
Musculoskeletal			
Muscle atrophy or weakness	28	0.34 (0.22, 0.48)	2.01 (0.74, 4.66), 0.132
Deforming or erosive arthritis (including reducible deformities, excluding avascular necrosis)	20	0.24 (0.15, 0.37)	0.81 (0.13, 2.83), 0.783
Osteoporosis with fracture or vertebral collapse (excluding avascular necrosis)	869	10.39 (9.75, 11.07)	1.46 (1.20, 1.77), <0.001
Avascular necrosis	47	0.56 (0.41, 0.75)	0.87 (0.30, 2.01), 0.773
Avascular necrosis (second event)	<5	–	0.87 (0.30, 2.01), 0.773
Osteomyelitis	28	0.34 (0.22, 0.48)	4.78 (2.17, 10.13), <0.001
Tendon rupture	46	0.55 (0.40, 0.73)	1.55 (0.67, 3.15), 0.263
Cutaneous			
Scarring chronic alopecia	51	0.61 (0.45, 0.80)	0.98 (0.37, 2.12), 0.958
Extensive scarring of the panniculum other than scalp and pulp space	<5	–	3.23 (2.47, 4.19), <0.001
Skin ulceration (excluding thrombosis) for >6 months	286	3.42 (3.04, 3.83)	3.23 (2.47, 4.19), <0.001
Gonadal			
Premature gonadal failure	<5	–	–^e^
Endocrine			
Diabetes mellitus	312	3.73 (3.34, 4.16)	2.16 (1.63, 2.83), <0.001
Malignancy			
Malignancy	701	8.38 (7.80, 9.00)	6.26 (5.27, 7.42), <0.001
Malignancy (second event)	13	0.16 (0.08, 0.27)	11.16 (3.70, 34.76), <0.001

Prevalence calculated for overall population, univariate logistic regressions performed in incident population only.

a Renal domain numbers are counts of items recorded (not as scored, due to weighting for end-stage renal disease).

b OR for renal score 1 = only eGFR <50% or proteinuria ≥3.5 g/24 h ever in record.

c OR for renal score 2 = eGFR <50% and proteinuria ≥3.5 g/24 h.

d OR for renal score 3 = end-stage renal disease (regardless of dialysis or transplantation).

e Unable to calculate.

Considering organ domains, musculoskeletal [*n* = 1002 (12%)], ocular [*n* = 853 (10.2%)] and cardiovascular [*n* = 781 (9.3%)] were the most involved domains. Endocrine [*n* = 312 (3.7%)], pulmonary [*n* = 126 (1.5%)] and gonadal (<1%) were the least commonly involved.

In the 5097 incident cases, average damage increased steadily, reaching a peak at year 20 (mean cohort eSDI 1.25) (see Supplementary Fig. S2, Supplementary File S1).

Univariate logistic regression was used to initially explore the relationship between individual items (and pre-specified covariates) with mortality in the total cohort (see Table 3 and Supplementary Table S3, Supplementary File S1). Most items increased the odds of mortality. For other items, some had a high point estimate but a wide CI. In addition, some items, e.g. within the skin domain, did not show an association with mortality. Older age at index and male sex were significantly associated with an increased odds of mortality. Results of the LASSO regression are shown in Supplementary Fig. S3, Supplementary File S1. Several baseline comorbidities (damage items recorded prior to diagnosis), including valvular disease, ulceration and pulmonary fibrosis, were shown to significantly increase the odds of mortality.

**Figure 1:**
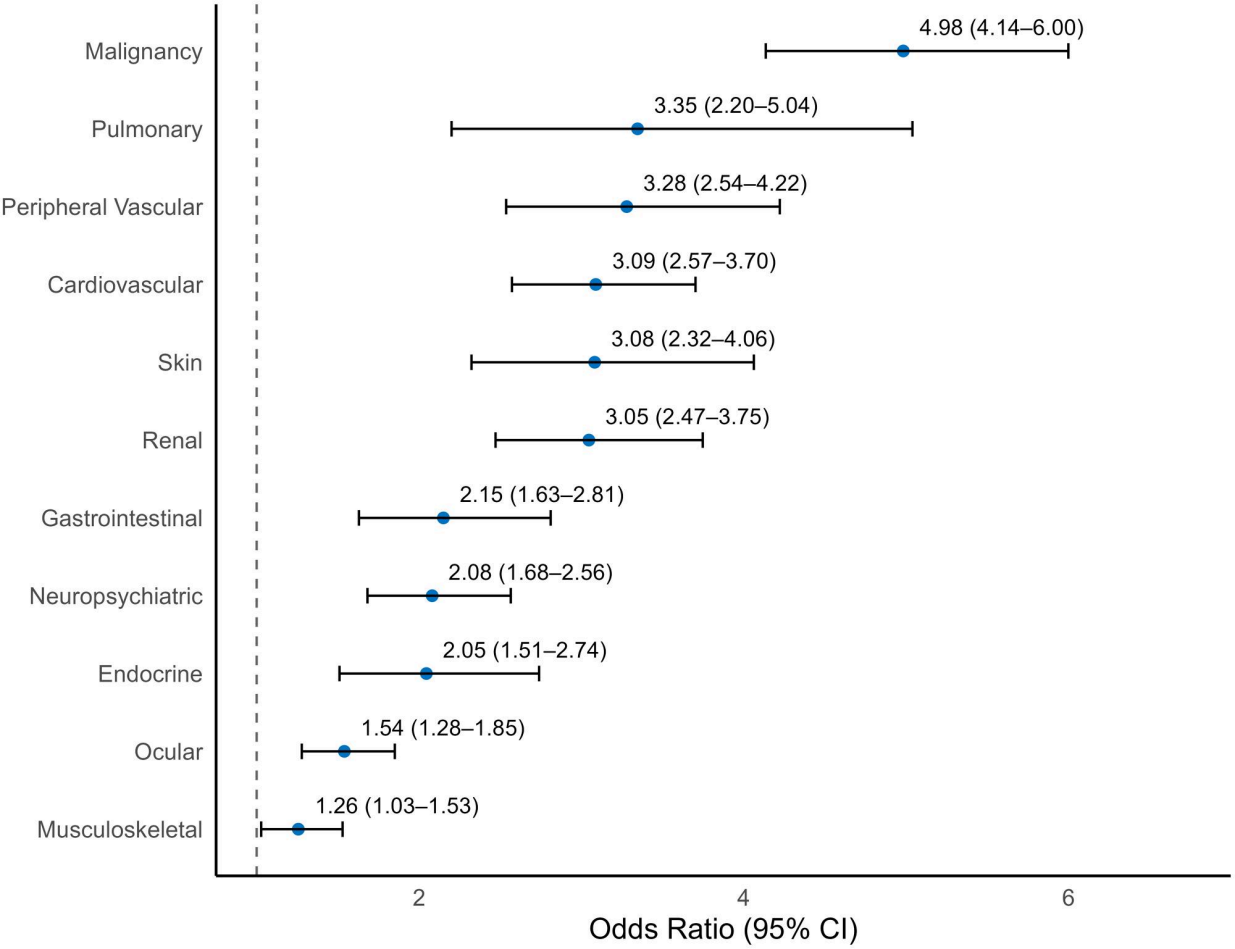
Odds of mortality with organ domain damage adjusted for sex and age at the index date. Vertical dashed line equal to an OR of 1

Fig. 1 shows the adjusted odds of mortality associated with the presence of damage in each organ domain. Damage across all organ systems was associated with increased odds of death. There was nearly a 5-fold increase in the odds of mortality seen with malignancy [OR 4.98 (95% CI 4.14, 6.00)]. Musculoskeletal damage showed the least impact but was still associated with a 26% increase in the odds of mortality [OR 1.26 (95% CI 1.03, 1.53)].

The results of the multivariable logistic regression model are presented in Supplementary Table S4, Supplementary File S1. Incurring damage ever was associated with an adjusted odds of mortality of 4.46 (95% CI 3.57, 5.57; *P* < 0.001).

**Figure 2:**
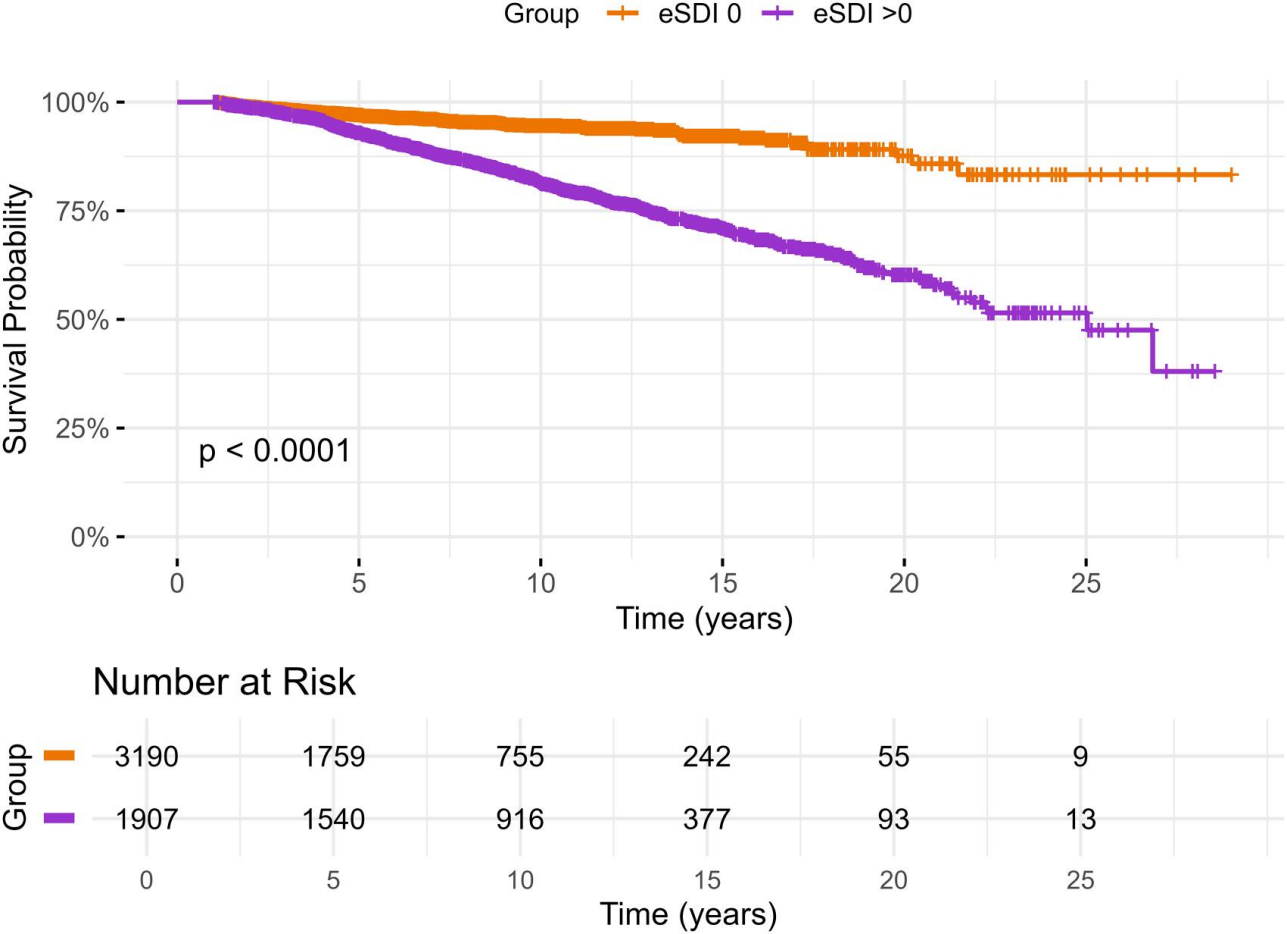
Kaplan–Meier analysis showing death-free survival probability stratified by damage. Time 0 in the risk table denotes the start of follow-up (index date) representing the number of incident patients at risk for death stratified by damage-ever status, irrespective of when damage occurred during follow-up

Of the incident cohort (*n* = 5097), 1907 (37.4%) developed damage (eSDI >0) over a mean of 8.14 years of follow-up (Fig. 2). The survival probability was lower for those with damage, with separation of the survival curves observed within 5 years of the index date. The larger reduction in the number of patients in the risk table in the eSDI = 0 group is reflective of more censoring in this group.

Both Cox models (SDI >0 *vs* SDI 0, and SDI as a continuous cumulative variable) showed a violation of the proportional hazard assumption for sex, which was therefore incorporated as strata, allowing the baseline hazard function to vary between males and females. Additionally, in the second model (damage as a continuous cumulative time varying covariate) using a step function for β(*t*) (different coefficients over separate time intervals) with 5-year intervals produced the best overall model fit.

Development of any damage (eSDI >0) over the follow-up was associated with a >10 times greater hazard of mortality [HR 11.77 (95% CI 8.22, 16.84), *P* < 0.001] (Supplementary Table S5 and Fig. S4, Supplementary File S1). Each additional year of age at the index date was associated with a 5% increase in the hazard of mortality. Smoking (ever) and non-White ethnicity were each associated with higher hazards of mortality (note both used imputed data). Several damage items recorded prior to diagnosis—e.g. myocardial infarction [HR 1.76 (95% CI 1.13, 2.74)] and malignancy [HR 1.44 (95% CI 1.08, 1.91)]—were also significantly associated with an increased hazard of mortality.

**Figure 3:**
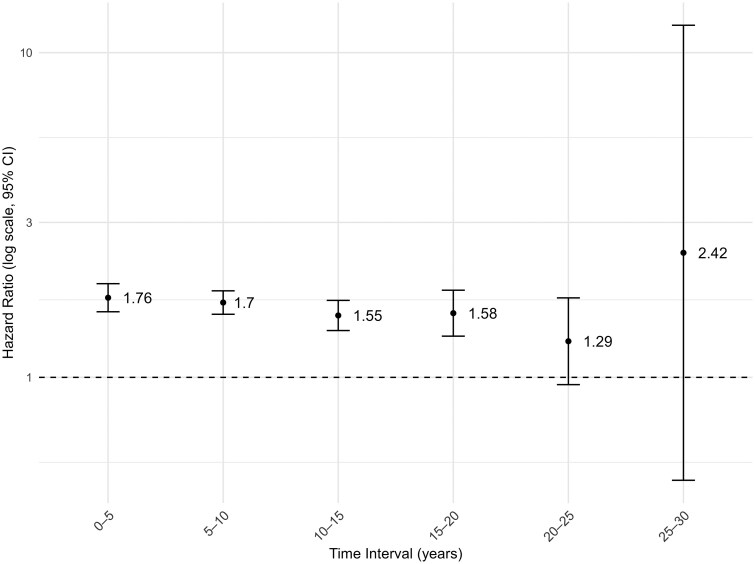
Hazard of mortality associated with cumulative damage compared with those with no damage by follow-up interval

Modelled as a cumulative continuous variable, higher SDI scores were associated with a greater hazard of mortality across the majority of follow-up (Fig. 3 and Supplementary Table S6, Supplementary File S1). The hazard of mortality varied differentially across the follow-up: 1.55 times (95% CI 1.39, 1.73; *P* < 0.001) at years 10–15 post-index and 1.76 times (95% CI 1.59, 1.94; *P* < 0.001) at years 0–5. Reference groups for HRs are eSDI = 0 in each time interval; e.g. each 1 unit increase in the damage score in years 0–5 is associated with a 76% greater hazard of mortality compared with no damage in that interval. HRs for year 20 onwards did not meet statistical significance, in the context of small patient numbers.

## Discussion

Our study has demonstrated that the measurement of SLE-based organ damage is feasible using routinely collected community-based data. Damage is common and associated with an increased risk of mortality.

It was possible to develop definitions for all items in the SDI using a modified eSDI except for shrinking lung syndrome (SLS), known to be an uncommon manifestation [13]. A recent study using a Swedish registry-based organ damage index (RBODI) also saw no occurrences of SLS [14]. Unlike our study, the Swedish study observed cases of pleural fibrosis, pulmonary infarct and chronic peritonitis, although the frequency was not reported. Reported prevalence of these items varies but is generally low [13, 15].

Few studies have reported the prevalence of all damage items; to our knowledge this is the first study to do this in this setting. Cataract, osteoporosis and malignancy were the most common individual items. Kallas *et al.* [16], reporting on the Hopkins Lupus Cohort over a mean follow-up of 13.5 years, also identified cataract and osteoporosis, alongside estimated glomerular filtration rate (eGFR) <50%, as the most common damage events. The prevalence of most items in the latter study was generally higher than in our study, reflective of their specialist lupus setting.

Twenty-six eSDI items had a frequency of <1%, commensurate with the literature [16]. Many items within the SDI are rare [3], and removal of redundant items may form part of ongoing efforts to update the index [17]. We measured some items, e.g. deforming arthritis (0.24%) at lower prevalences than anticipated. Gomez *et al.* [14] reported challenges with similar items in their RBODI; comparing the RBODI to traditional chart SDI assessment, the musculoskeletal domain had the lowest negative predictive value [NPV 0.86 (95% CI 0.81, 0.9)], suggestive of an underestimation of these items. We took a restrictive approach to item identification, favouring strict definitions to limit false positives. Chosen codes were double reviewed by two clinicians; those chosen were deemed to best represent true damage. This approach may mean some damage has not been identified, particularly true for items favouring clinical assessment (e.g. muscle atrophy) and uncommon items (e.g. chronic peritonitis). External validation of the eSDI, as performed by Gomez *et al.* [14], could provide a means to further interrogate the validity of the selected codes.

Musculoskeletal, ocular and cardiovascular were the most frequently affected organ domains. Organ-level damage has been reported more comprehensively than individual items. Musculoskeletal damage is the most common form of damage across a variety of geographical settings, including Asia [18], North America [19] and Europe [20], with a reported prevalence between 14% [21] and 54.7% [22]. Multiple cohorts have reported ocular damage as either the first or second most commonly affected domain, with a reported prevalence between 7% [23] and 31.5% [22]. Cardiovascular damage was relatively common in our study compared with others [23]. Cardiovascular damage may accrue later in the disease course and is thought to reflect accelerated atherosclerosis consequential to the cumulative inflammatory burden [22]. A longer follow-up duration in our study compared with others [23] may have allowed time for cardiovascular disease to develop. Additionally, cardiovascular damage has been shown to be more prominent in later-onset SLE [24, 25]. Our study population was relatively old (mean age at index 46.7 years), in keeping with other registry studies of SLE [14, 26], but different from hospital-based cohorts. Therefore, differences in our underlying SLE population *vs* those comprising more traditional cohorts may also underlie these differential damage patterns.

We have confirmed that the association of damage with increased mortality found in more specialist settings is also replicated in a primary care setting. The link between the SDI and mortality has been a key marker of the index’s construct validity [4]. Replicating this association using the eSDI reinforces its credibility as a measurement tool. In the Grupo Latino Americano De Estudio del Lupus (GLADEL) cohort, an SDI of 1 compared with 0 imparted 2.8 times greater odds of mortality (CI 1.2, 6.4) [27]. Manger *et al.* [28] explored the effect of a change in SDI, observing that an SDI increase ≥2 conferred 7.7 times greater odds of mortality. A systematic literature review summarising 14 studies evaluating the SDI as a continuous predictor of mortality observed a 34% increased risk of death for each 1 point increase in the SDI score [5]. In our study we also found that higher SDI scores are associated with a higher hazard of death. We observed variation in the magnitude of this hazard risk over time, which other studies have not reported. In contrast to several studies in Murimi-Worstell *et al.*’s aforementioned review we modelled damage as a time-varying covariate from diagnosis rather than measuring at fixed time points post-diagnosis (e.g. 1 year) [29], as well as incorporating baseline comorbidities. Small patient numbers at the limits of follow-up duration may account for instability in some of our later estimates.

Our study has several strengths. The use of electronic healthcare data has allowed for the assessment of damage in the largest UK SLE population to date, providing a comprehensive overview of its prevalence in a novel setting. A long study period has enabled observation of trends over time and testing of the expected association with mortality.

Certain damage items were challenging to define, particularly for rarer manifestations. As such, it is probable that some damage events have not been captured. However, many of the items (e.g. myocardial infarction) in the damage index are common in the general population and the validity of their CPRD coding was tested. It seems a reasonable assumption that these items have been well captured. Furthermore, any misclassification is more likely to under- rather than overestimate the strength of the associations with mortality, suggesting these results are likely to remain significant.

In line with the current SDI framework, damage items recorded prior to diagnosis were classified as comorbidities rather than damage. Many individuals in our study had these items recorded prior to the index date. This aligns with other registry data where comorbidities are increased prior to diagnosis [30]. Several conditions (e.g. pulmonary fibrosis) were associated with mortality on regression analysis. It is now widely recognised that some individuals will incur SLE-related damage prior to formal diagnosis. Typically this occurs in the context of evolution from other autoimmune diseases or diagnostic delay. This could suggest the eSDI, and perhaps therefore the SDI, is not capturing the full extent of damage, potentially underestimating its relationship with mortality. Damage events deemed attributable to SLE prior to diagnosis are likely to be included in the updated index [17].

There was a significant amount of missing ethnicity data (>50%). Although missingness was addressed with imputation before incorporation into our models, any associations with ethnicity reported here should be interpreted cautiously. Future work looking at patterns of damage would benefit from obtaining fuller demographic data to help better understand this relationship. We did not have information relating to certain aspects of disease, such as disease activity, and it is probable that other influences on damage have not been fully captured. For example, low counts of some medications (e.g. belimumab) likely represent underrecording of these medications in primary care. Unmeasured confounding could mean the association between damage and mortality has been overestimated. However, our results are concordant with the established literature, showing damage does impact mortality, making it unlikely that an inaccurate association has been observed.

In conclusion, this study demonstrates that measuring SLE-related organ damage using routinely collected primary care healthcare data is feasible. Our findings support the validity of register-based approaches to damage assessment, highlighting the utility of real-world data in understanding long-term outcomes in large unselected SLE populations. Crucially, damage was consistently associated with mortality, emphasising that all clinicians caring for SLE patients should be vigilant for damage development. Further work interrogating damage patterns in primary care settings may yield important insights into the contemporary outlook of SLE.

## Supplementary Material

rkag079_Supplementary_Data

## Data Availability

Data underlying this article are provided by the CPRD under licence. Data will be shared on request to the corresponding author with permission of the CPRD. Codes and algorithms utilised in this study are available via Mendeley Data (doi: 10.17632/zwhzfggvf9.1).

## References

[1] Yen EY , Shaheen M, Woo JMP et al 46-year trends in systemic lupus erythematosus mortality in the United States, 1968 to 2013: a nationwide population-based study. Ann Intern Med 2017;167:777–85. 29086801 10.7326/M17-0102 PMC6188647

[2] Dayal N , Gordon C, Tucker L, Isenberg D. The SLICC damage index: past, present and future. Lupus 2002;11:261–5. 12043892 10.1191/0961203302lu190sa

[3] Barber MR , Johnson SR, Gladman DD, Clarke AE, Bruce IN. Evolving concepts in systemic lupus erythematosus damage assessment. Nat Rev Rheumatol 2021;17:307–8. 33859399 10.1038/s41584-021-00611-4

[4] Gladman D , Ginzler E, Goldsmith C et al The development and initial validation of the Systemic Lupus International Collaborating Clinics/American College of Rheumatology damage index for systemic lupus erythematosus. Arthritis Rheum 1996;39:363–9. 8607884 10.1002/art.1780390303

[5] Murimi-Worstell IB , Lin DH, Nab H et al Association between organ damage and mortality in systemic lupus erythematosus: a systematic review and meta-analysis. BMJ Open 2020;10:e031850. 10.1136/bmjopen-2019-031850 PMC7247371 32444429

[6] Clinical Practice Research Datalink. Primary care and linked data to address public health research. London: Medicines and Healthcare Products Regulatory Agency, 2022.

[7] Clinical Practice Research Datalink. CPRD GOLD January 2021 dataset. London: Medicines and Healthcare Products Regulatory Agency, 2023.

[8] Ellis J , McHugh N, Pauling JD et al Changes in the incidence and prevalence of systemic lupus erythematosus between 1990 and 2020: an observational study using the Clinical Practice Research Datalink (CPRD). Lupus Sci Med 2024;11:e001213. 39067871 10.1136/lupus-2024-001213 PMC11284910

[9] Rees F , Doherty M, Grainge M et al The incidence and prevalence of systemic lupus erythematosus in the UK, 1999–2012. Ann Rheum Dis 2016;75:136–41. 25265938 10.1136/annrheumdis-2014-206334 PMC4717400

[10] Langham J , Barut V, Samnaliev M et al Disease severity, flares and treatment patterns in adults with systemic lupus erythematosus in the UK: a real-world observational retrospective cohort analysis. Rheumatol Adv Pract 2021;5:rkab061. 34557623 10.1093/rap/rkab061 PMC8452998

[11] Health Data Research UK. HDR UK phenotype library. London: Health Data Research UK, 2023.

[12] Bruce IN , O’Keeffe AG, Farewell V et al Factors associated with damage accrual in patients with systemic lupus erythematosus: results from the Systemic Lupus International Collaborating Clinics (SLICC) inception cohort. Ann Rheum Dis 2015;74:1706–13. 24834926 10.1136/annrheumdis-2013-205171 PMC4552899

[13] Little JC. Glucocorticoid use and its association with damage accrual in the systemic lupus international collaborating clinics inception cohort. Master’s thesis, University of Manchester, Manchester, UK, 2017.

[14] Gomez A , Parodis I, Saleh M et al Development and evaluation of a register-based organ damage index in systemic lupus erythematosus: a nationwide, population-based study from Sweden. Lupus Sci Med 2025;12:e001403. 40011068 10.1136/lupus-2024-001403 PMC11865802

[15] Kallas R , Li J, Petri M. Association of African-American ethnicity and smoking status with total and individual damage index in systemic lupus erythematosus. Clin Rheumatol 2020;39:365–73. 31705325 10.1007/s10067-019-04800-1 PMC7340111

[16] Kallas R , Li J, Goldman DW, Magder LS, Petri M. Trajectory of damage accrual in systemic lupus erythematosus based on ethnicity and socioeconomic factors. J Rheumatol 2022;49:1229–35. 35914791 10.3899/jrheum.211135

[17] Johnson SR , Gladman DD, Brunner HI et al Evaluating the construct of damage in systemic lupus erythematosus. Arthritis Care Res (Hoboken) 2023;75:998–1006. 34962100 10.1002/acr.24849

[18] Kandane-Rathnayake R , Milea D, Louthrenoo W et al Longitudinal associations of flare and damage accrual in patients with systemic lupus erythematosus. Lupus Sci Med 2025;12:e001363. 39832908 10.1136/lupus-2024-001363 PMC11751792

[19] Watson P , Brennan A, Birch H, Fang H, Petri M. An integrated extrapolation of long-term outcomes in systemic lupus erythematosus: analysis and simulation of the Hopkins lupus cohort. Rheumatology (Oxford) 2015;54:623–32. 25234659 10.1093/rheumatology/keu375

[20] Altabás-González I , Rua-Figueroa I, Mouriño C et al Damage in a large systemic lupus erythematosus cohort from the Spanish Society of Rheumatology Lupus Registry (RELESSER) with emphasis on the cardiovascular system: a longitudinal analysis. Lupus Sci Med 2024;11:e001064. 39097409 10.1136/lupus-2023-001064 PMC11331961

[21] Artim-Esen B , Şahin S, Çene E et al Comparison of disease characteristics, organ damage, and survival in patients with juvenile-onset and adult-onset systemic lupus erythematosus in a combined cohort from 2 tertiary centers in Turkey. J Rheumatol 2017;44:619–25. 28298568 10.3899/jrheum.160340

[22] Gladman DD , Urowitz MB, Rahman P, Ibañez D, Tam LS. Accrual of organ damage over time in patients with systemic lupus erythematosus. J Rheumatol 2003;30:1955–9. 12966597

[23] Toloza SMA , Roseman JM, Alarcón GS et al Systemic lupus erythematosus in a multiethnic US cohort (LUMINA): XXII. Predictors of time to the occurrence of initial damage. Arthritis Rheum 2004;50:3177–86. 15476246 10.1002/art.20578

[24] Maddison P , Farewell V, Isenberg D et al The rate and pattern of organ damage in late-onset systemic lupus erythematosus. J Rheumatol 2002;29:913–7. 12022349

[25] Bertoli AM , Alarcón GS, Calvo-Alén J et al Systemic lupus erythematosus in a multiethnic US cohort: clinical features, course, and outcome in patients with late-onset disease. Arthritis Rheum 2006;54:1580–7. 16645994 10.1002/art.21765

[26] Schultze M , Garal-Pantaler E, Pignot M et al Clinical and economic burden of organ damage among patients with systemic lupus erythematosus in a real-world setting in Germany. BMC Rheumatol 2024;8:18. 38755673 10.1186/s41927-024-00387-6 PMC11100138

[27] Pons-Estel BA , Catoggio LJ, Cardiel MH et al The GLADEL multinational Latin American prospective inception cohort of 1,214 patients with systemic lupus erythematosus: ethnic and disease heterogeneity among “Hispanics”. Medicine (Baltimore) 2004;83:1–17. 14747764 10.1097/01.md.0000104742.42401.e2

[28] Manger K , Manger B, Repp R et al Definition of risk factors for death, end stage renal disease, and thromboembolic events in a monocentric cohort of 338 patients with systemic lupus erythematosus. Ann Rheum Dis 2002;61:1065–70. 12429536 10.1136/ard.61.12.1065 PMC1753955

[29] Lopez R , Davidson JE, Beeby MD, Egger PJ, Isenberg DA. Lupus disease activity and the risk of subsequent organ damage and mortality in a large lupus cohort. Rheumatology (Oxford) 2012;51:491–8. 22109798 10.1093/rheumatology/ker368

[30] Hansen RB , Simard JF, Faurschou M, Jacobsen S. Distinct patterns of comorbidity prior to diagnosis of incident systemic lupus erythematosus in the Danish population. J Autoimmun 2021;123:102692. 34364172 10.1016/j.jaut.2021.102692

