# Supplementary material for: The SLICC/ACR damage index, assessed using a community-based electronic healthcare record, predicts mortality in SLE patients: results from the United Kingdom Clinical Practice Research Datalink
Source: Rheumatol Adv Pract. 2026 Jul 13;10(3):rkag079. doi: 10.1093/rap/rkag079 (PMC13405242; doi:10.1093/rap/rkag079)
Supplement: rkag079_Supplementary_Data [file rkag079_supplementary_data.zip › Supplementary_File_1.docx]

**Supplementary File 1**

**Supplementary Figures**

[Supplementary Figure S1 Study Design Diagram 3](#_Toc231991814)

[Supplementary Figure S2 Average SDI Score and Number in the Incident SLE Cohort by Year of Follow Up 4](#_Toc231991815)

[Supplementary Figure S3 LASSO Regression Analysis for Identification of Mortality Predictors 5](#_Toc231991816)

[Supplementary Figure S4 Extended Cox Proportional Hazards Model Showing the Association Between Any Damage (eSDI>0) and Mortality 6](#_Toc231991817)

**Supplementary Tables**

[Supplementary Table S1 Statistical Packages 7](#_Toc231991818)

[Supplementary Table S2 Damage Item Definitions 9](#_Toc231991819)

[Supplementary Table S3 Univariate Logistic Regression of Association Between Co-Variates with Mortality 13](#_Toc231991820)

[Supplementary Table S4 Multivariable Logistic Regression Analysis of the Association Between Damage Items and Mortality Outcomes 14](#_Toc231991821)

[Supplementary Table S5 Extended Cox Proportional Hazards Model Showing Measured Association Between any Damage (eSDI>0) and Mortality 15](#_Toc231991822)

[Supplementary Table S6 Extended Cox Proportional Hazards Model Showing Measured Association Between any Cumulative Damage and Mortality 16](#_Toc231991823)

Item definitions


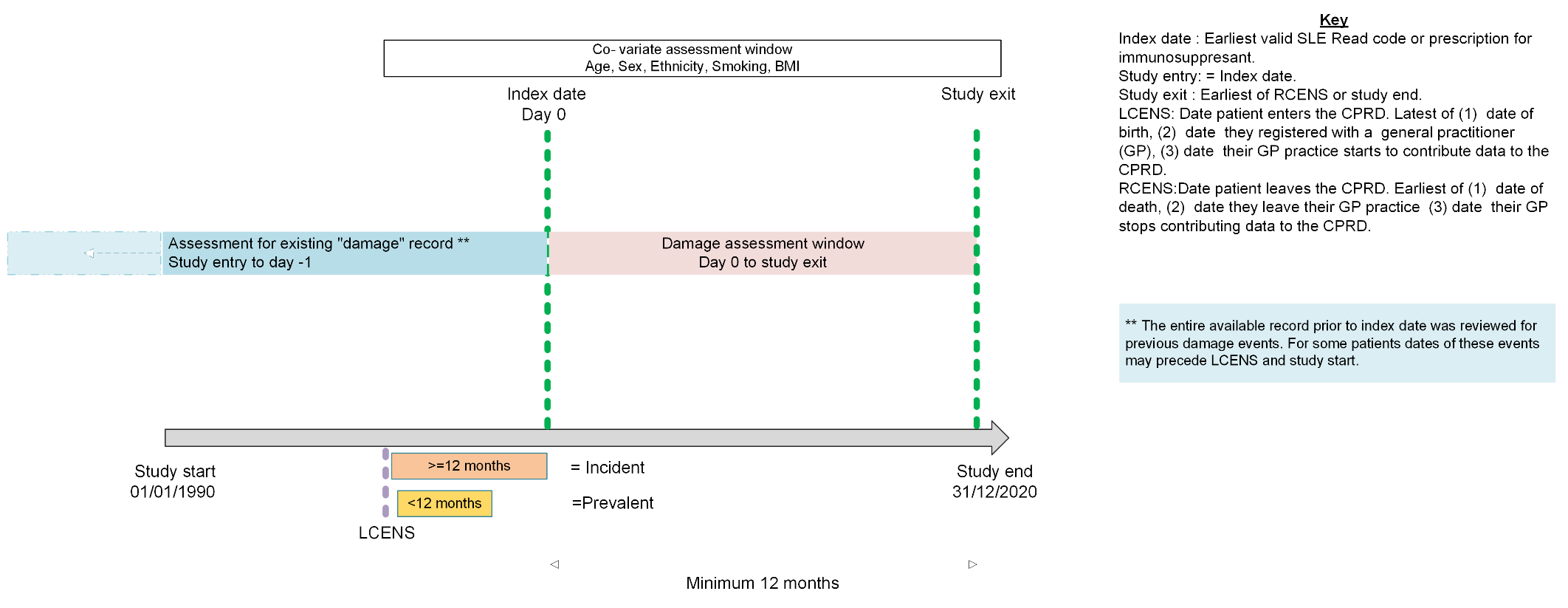


Supplementary Figure S1 Study Design Diagram

Figure 1 Study design diagram

**
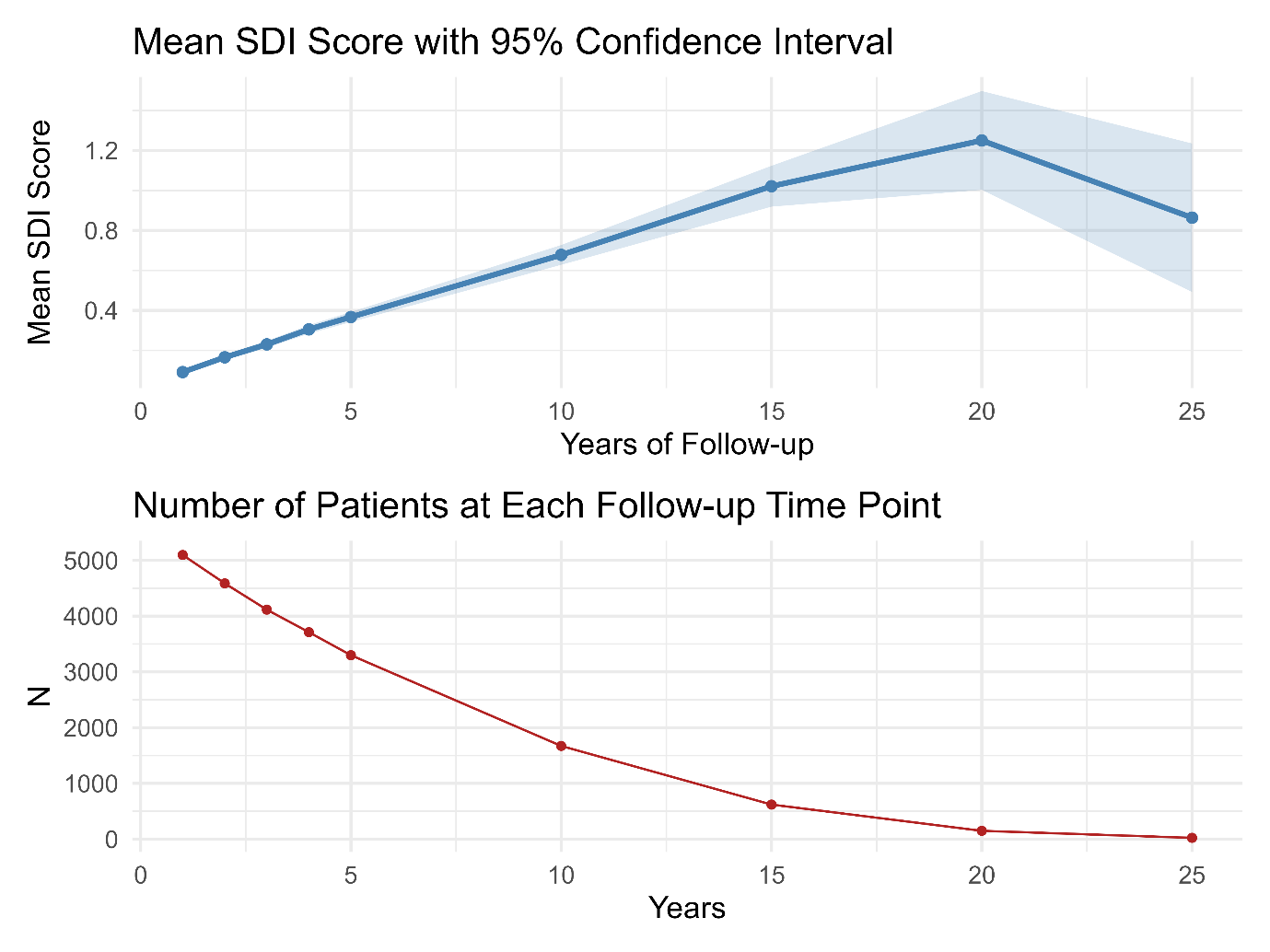
**

Supplementary Figure S2 Average SDI Score and Number in the Incident SLE Cohort by Year of Follow Up

**
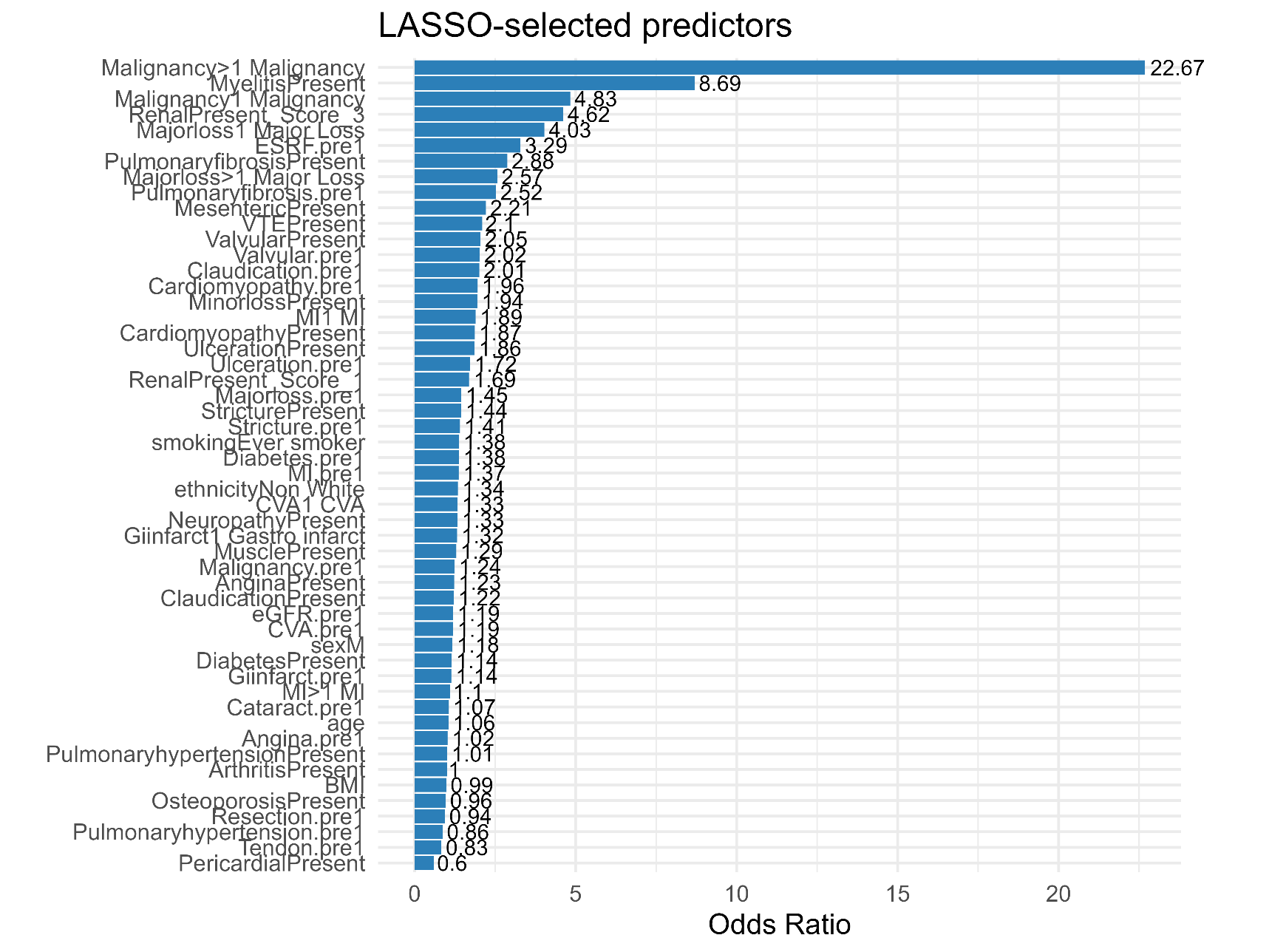
**

Supplementary Figure S3 LASSO Regression Analysis for Identification of Mortality Predictors

**Key:**

**>1 for items with multiple events**

**.pre identifies a damage event condition prior to index**

**Smoking and ethnicity imputed variables.**

**Odds Ratios represent the average pooled exponentiated coefficients across all imputed data**


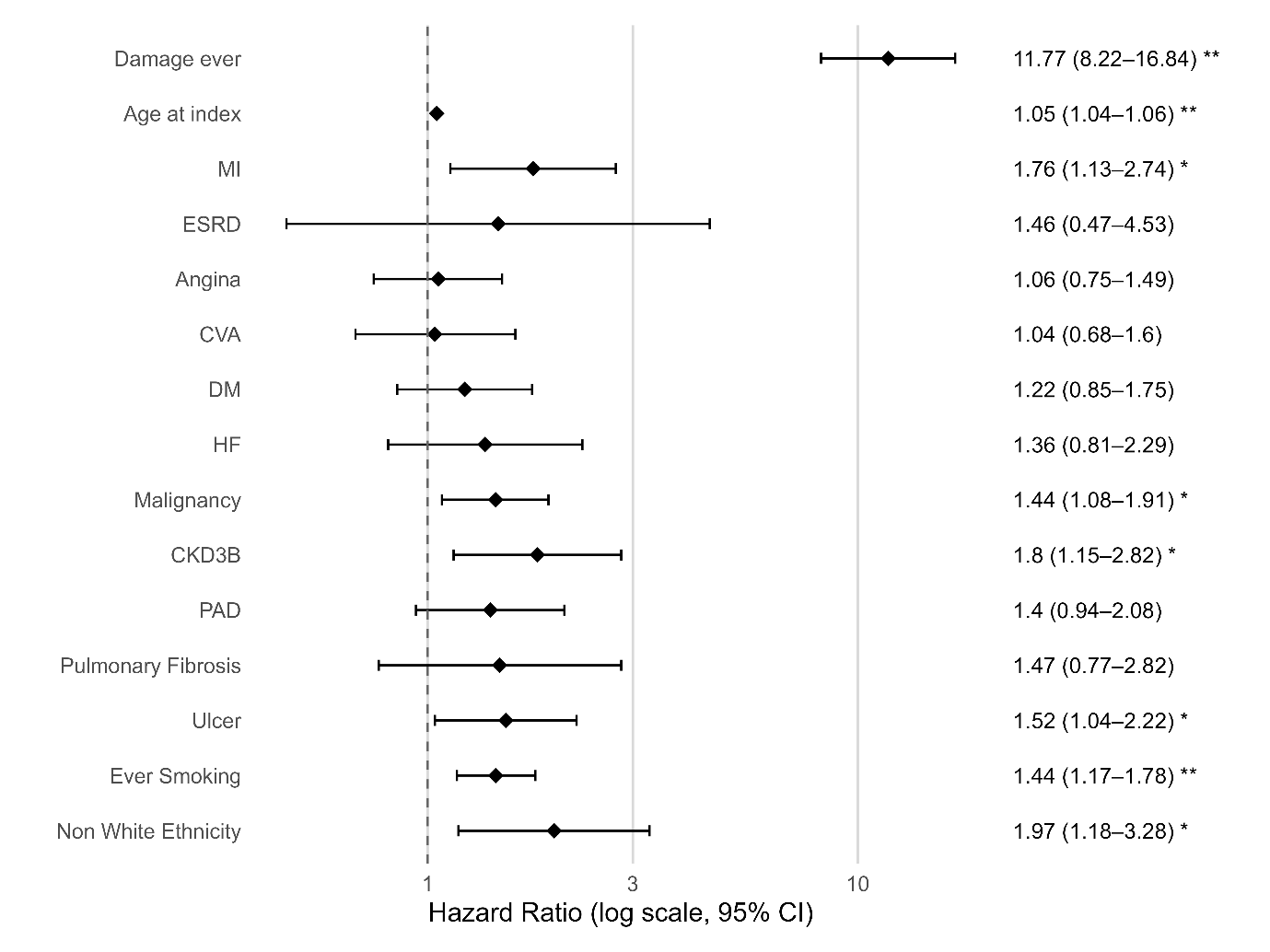


Supplementary Figure S4 Extended Cox Proportional Hazards Model Showing the Association Between Any Damage (eSDI>0) and Mortality

***p value <0.05,** p value <0.001**

Supplementary Table S1 Statistical Packages

| **Package** | **Version** | **Depends** |
| --- | --- | --- |
| Epi | 2.59 | R (>= 3.5.0), utils |
| GGally | 2.2.1 | R (>= 3.1), ggplot2 (>= 3.4.4) |
| Polychrome | 1.5.4 | R (>= 4.4) |
| RColorBrewer | 1.1-3 | R (>= 2.0.0) |
| ResourceSelection | 0.3-6 | R (>= 2.13.0) |
| VIM | 6.2.2 | R (>= 3.5.0),colorspace,grid |
| binom | 1.1-1.1 | NA |
| broom | 1.0.7 | R (>= 3.5) |
| car | 3.1-3 | R (>= 3.5.0), carData (>= 3.0-0) |
| caret | 7.0-1 | ggplot2, lattice (>= 0.20), R (>= 3.2.0) |
| data.table | 1.17.4 | R (>= 3.3.0) |
| dplyr | 1.1.4 | R (>= 3.5.0) |
| emmeans | 1.11.1 | R (>= 4.1.0) |
| epitools | 0.5-10.1 | R (>= 2.10) |
| finalfit | 1.0.8 | NA |
| forcats | 1.0.0 | R (>= 3.4) |
| ggpattern | 1.1.4 | NA |
| ggplot2 | 3.5.2 | R (>= 3.5) |
| glmnet | 4.1-8 | R (>= 3.6.0), Matrix (>= 1.0-6) |
| gridExtra | 2.3 | NA |
| here | 1.0.1 | NA |
| lubridate | 1.9.4 | methods, R (>= 3.2) |
| mice | 3.17.0 | R (>= 2.10.0) |
| mitools | 2.4 | NA |
| patchwork | 1.3.0 | NA |
| readr | 2.1.5 | R (>= 3.6) |
| scales | 1.3.0 | R (>= 3.6) |
| survival | 3.7-0 | R (>= 3.5.0) |
| survminer | 0.5.0 | ggplot2(>= 3.4.0), ggpubr(>= 0.1.6) |
| tidyr | 1.3.1 | R (>= 3.6) |
| tidyverse | 2.0.0 | R (>= 3.3) |
| writexl | 1.5.2 | NA |

**SDI Item definitions**

Supplementary Table 1 gives an overview of the mechanisms used for identification of different damage items in the CPRD. This does not encompass all details e.g. data cleaning processes, test range thresholds ( available on request) but provides a summary of the approach followed. Other general principles applied, are outlined below:

1. Damage dates
   1. In general, the earliest item code recorded ≥ index date has been used as the date of first damage.
2. Acute vs chronic codes
   1. Where available codes implying chronicity have been used. For some items (e.g. scarring alopecia) these were limited. In these instances, acute codes have also been used. Where this has occurred 2 codes ≥ 6 months (182.6 days) apart were required to be classed as damage (second date taken as damage date).
3. Items with multiple possible conditions
   1. Some items span different conditions e.g. cognitive impairment includes dementia and psychosis. Where this occurs, these were individually categorised if the same form was present pre and post index is was not scored as damage.
      1. Example 1: psychosis coded pre index, dementia post index -> patient scored as cognitive impairment damage.
      2. Example 2: psychosis coded pre index psychosis post index -> patient **not** scored as cognitive impairment damage.
      3. Example 3: psychosis and dementia coded pre index, psychosis and dementia post index -> patient **not** scored as cognitive impairment damage.
   2. Items where this has been factored into scoring are highlighted with a * in the column “*not scored damage if evidence of existing pre diagnosis*”.
4. Multiple code types
   1. For some items multiple different types of code have been used to identify items e.g. diabetes. Where this occurs:
      1. Read and test data code: in general, the earliest of either method ≥ index date has been taken as the damage date
      2. Read and prescription code: in general, the earliest of either method ≥ index date has been taken as the damage date.
      3. Exception to this is items specifying there must be a prescription to score as damage e.g. seizure, in which case the prescription code has been used as the damage date.
5. Paired reviews
   1. Preliminary code lists were devised by the lead author (JE). Paired reviews were undertaken to decide which codes to include. Reviewers were asked to retain codes which 1) Accurately represented the damage item and 2) Represented damage (i.e. permanent change). Disagreements between reviewers were settled by discussion, or where not possible via adjudication from another investigator (SS).

Supplementary Table S2 Damage Item Definitions

| Item | Read (diagnostic and/ or procedural) code | Acute code utilised | Prescription code | Test data | Not scored if present pre diagnosis | Comments |
| --- | --- | --- | --- | --- | --- | --- |
| Any cataract ever | ✓ |  |  |  | ✓ |  |
| Retinal change or optic atrophy | ✓ |  |  |  | ✓ |  |
| Cognitive impairment | ✓ | ✓ |  |  | ✓* |  |
| Seizures requiring therapy for 6 months | ✓ |  | ✓ |  | ✓ | Patients were required to have ≥ 2 codes for a seizure medication separated by ≥ 6 months (182.6 days) for scoring as damage. The second of the two codes was taken as the date of the damage event. |
| Cerebrovascular accident or resection (not for malignancy) ever | ✓ |  |  |  |  | Resection events were reviewed for the presence of a cranial malignancy code within +/-365 days of the resection code. This window could cross the index date. Where present this was deemed a resection due to malignancy and therefore excluded. |
| Cerebrovascular accident ever (2nd event) | ✓* |  |  |  |  | Scored for a second cerebral vascular accident if it occurs≥ 6 months (≥182.6 days) after the other recorded event. |
| Cranial or peripheral neuropathy (excluding optic) | ✓ |  |  |  |  |  |
| Transverse myelitis | ✓ |  |  |  | ✓ |  |
| Estimated or measured glomerular filtration rate <50% *** | ✓ |  |  | ✓ | ✓ | If test data used, 2 x eGFR (recorded or calculated) ≤ 45 ml/min/1.73m^2^ and >15 ml/min/1.73m^2 ≥^90 days apart required, later of the two dates taken as damage date.  Read code specific to stage 3B or 4 CKD. |
| Proteinuria ≥3.5 gm/24 hours | ✓ |  |  | ✓ | ✓ | ENTTYPE only available for urine albumin: creatinine ratio, transformed to match protein: creatinine ratio (PCR) as per SDI.  Read codes for nephrotic syndromes. |
| End-stage renal disease (regardless of dialysis or transplantation) (ESRD) | ✓ |  |  | ✓ | ✓ | If test data used, 2 x eGFR (recorded or calculated) ≤ 15 ml/min/1.73m^2^ ≥90 days apart required, later of the two dates taken as damage date.  Read code specific to stage ESRD and or renal transplant. |
| Pulmonary hypertension | ✓ |  | ✓ |  | ✓ | Only Pulmonary Hypertension specific prescriptions used. |
| Pulmonary fibrosis | ✓ |  |  |  | ✓ |  |
| Shrinking lung | N/A | N/A | N/A | N/A | N/A | Not possible to identify |
| Pleural fibrosis | ✓ |  |  |  | ✓ | Only one Read code (H51y600, Fibrothorax) which specifically references pleural fibrosis. Codes for other types of fibrotic change e.g. pleural plaque excluded as felt to be non- specific. |
| Pulmonary infarction (or resection not for malignancy) | ✓ |  |  |  |  | Resection events were reviewed for the presence of a pulmonary malignancy code within +/- 365 days of the resection code. This window could cross the index date. Where present this was deemed a resection due to malignancy and therefore excluded. |
| Angina or coronary artery bypass | ✓ |  |  |  | ✓ | Different definitions (with inclusion/ exclusion) of procedural codes tested, with no significant difference in prevalence counts. |
| Myocardial infarction (MI)ever | ✓ |  |  | ✓ |  | Read codes deemed as definite MI no test data required. For probable codes presence of test code associated with MI e.g. Troponin within +/- 30 days of MI code also required.  Different definitions (with inclusion/exclusion) of codes suggestive of MI complication tested, with no significant difference in prevalence counts including these. |
| Myocardial infarction ever (2nd event) | ✓ |  |  | ✓ |  | Scored for a second myocardial infarction if it occurs≥ 6 months (≥182.6 days) after the other recorded event. |
| Cardiomyopathy | ✓ |  |  |  | ✓ |  |
| Valvular disease | ✓ |  |  |  | ✓* | Different definitions (with inclusion/ exclusion) of procedural codes tested, with no significant difference in prevalence counts. |
| Pericarditis for 6 months, or pericardiectomy | ✓ |  |  |  | ✓ |  |
| Claudication for 6 months | ✓ |  |  |  | ✓ |  |
| Minor tissue loss (pulp space) | ✓ |  |  |  |  |  |
| Significant tissue loss ever | ✓ |  |  |  |  |  |
| Significant tissue loss ever (2nd event) | ✓ |  |  |  |  | Scored for a second tissue loss event if it occurs≥ 6 months (≥182.6 days) after the last recorded event. |
| Venous thrombosis (VTE) with swelling, ulceration, or venous stasis | ✓ |  |  |  |  | Read codes for lower limb VTE also required code suggesting presence of complication e.g. leg ulcer post VTE code. Procedure codes e.g. thrombectomy no additional evidence required. Non lower limb VTE e.g. pulmonary embolus no additional evidence required (not possible to accurately identify additional evidence for each site, and non-limb VTE taken as more significant). |
| Infarction or resection of bowel below duodenum, spleen, liver, or gall bladder ever, for cause any | ✓ |  |  |  | ✓* |  |
| Infarction or resection of bowel below duodenum, spleen, liver, or gall bladder ever, for cause any (2nd event) | ✓ |  |  |  | ✓* | Scored for a second event if it occurs≥ 6 months (≥182.6 days) after the other recorded event. |
| Mesenteric insufficiency | ✓ |  |  |  | ✓ |  |
| Chronic peritonitis | ✓ |  |  |  | ✓ |  |
| Stricture or upper gastrointestinal tract surgery ever | ✓ |  |  |  |  |  |
| Pancreatitis: insufficiency requiring enzyme replacement or with pseudocyst | ✓ |  | ✓ |  | ✓ | If code for chronic pancreatitis used (rather than pseudocyst) require 2 Read codes with ≥ 6 months (182.6 days) between codes. |
| Muscle atrophy or weakness | ✓ |  |  |  |  |  |
| Deforming or erosive arthritis (including reducible deformities, excluding avascular necrosis) | ✓ |  |  |  |  |  |
| Osteoporosis with fracture or vertebral collapse (excluding avascular necrosis) | ✓ |  |  |  | ✓ | Either Read code specifically for osteoporotic fracture or read code for osteoporosis and read code for fracture deemed consistent with a fragility fracture. |
| Avascular necrosis | ✓ |  |  |  |  |  |
| Avascular necrosis (2nd event) | ✓ |  |  |  |  | Scored for a second event if it occurs≥ 6 months (≥182.6 days) after the other recorded event. |
| Osteomyelitis | ✓ |  |  |  |  |  |
| Tendon rupture | ✓ |  |  |  |  |  |
| Scarring chronic alopecia | ✓ | ✓ |  |  | ✓ | No existing record of scarring alopecia. |
| Extensive scarring of panniculum other than scalp and pulp space | ✓ | ✓ |  |  |  |  |
| Skin ulceration (excluding thrombosis) for >6 months | ✓ | ✓ |  |  |  | Must have no record or lower limb VTE within +/-365 days of code for leg ulceration |
| Premature gonadal failure | ✓ |  |  |  |  | Only assessed in females < age 40. |
| Diabetes (DM) | ✓ |  | ✓ | ✓ | ✓ | In isolation code suggestive of definite treated diabetes (e.g. insulin dependant diabetes, type 1 diabetes) or diabetes code and drug code for diabetic medication or test code suggestive of diabetes (fasting glucose or HBA1c) and code for diabetic medication. |
| Malignancy | ✓ |  |  |  | ✓ | Malignancies categorised by site e.g. thyroid, renal, CNS to mitigate risk of scoring same malignancy twice. Note several malignancy codes unable to define location “unclassifiable”. |
| Malignancy (2nd event) | ✓ |  |  |  | ✓ |  |

Supplementary Table S3 Univariate Logistic Regression of Association Between Co-Variates with Mortality

|  | SLE | |
| --- | --- | --- |
| Explanatory variable* | OR | (95% CI, p value) |
| Age at index (years) | 1.07 | (1.07-1.08, p<0.001) |
| Male sex | 2.01 | (1.70-2.38, p<0.001) |
| Index year | 0.94 | (0.93-0.95, p<0.001) |
| Disease duration (years) | 0.98 | (0.97-0.99, p<0.001) |

Supplementary Table S4 Multivariable Logistic Regression Analysis of the Association Between Damage Items and Mortality Outcomes

| **Explanatory variable** | **OR (95% CI)**  **(Univariable)** | **OR (95% CI)**  **(Multivariable)** |
| --- | --- | --- |
| Age at index | 1.08 (1.07-1.08) | 1.06 (1.05-1.07) |
| Damage ever (eSDI>0) | 6.83 (5.56-8.44) | 4.46 (3.57-5.57) |
| Male sex | 1.92 (1.54-2.39) | 1.40 (1.08-1.80) |
| Ever smoker* | 1.49 (1.24-1.79) | 1.62 (1.29-2.03) |
| All other ethnic groups combined (excluding White ethnicity)* | 0.68 (0.36-1.17) | 1.79 (0.96-3.33) |
| Baseline CVA | 3.03 (1.92-4.65) | 1.55 (0.92-2.60) |
| Baseline CKD3B | 5.67 (3.55-8.94) | 1.39 (0.79-2.44) |
| Baseline ESRD | 7.03 (2.02-23.42) | 3.25 (0.69-15.25) |
| Baseline Angina | 4.74 (3.39-6.55) | 1.09 (0.72-1.65) |
| Baseline MI | 5.21 (3.30-8.09) | 1.72 (0.97-3.06) |
| Baseline Heart failure | 9.10 (4.95-16.75) | 1.76 (0.82-3.75) |
| Baseline PAD | 8.20 (5.15-13.03) | 2.23 (1.30-3.83) |
| Baseline DM | 3.58 (2.48-5.08) | 1.35 (0.87-2.08) |
| Baseline Malignancy | 3.02 (2.29-3.95) | 1.37 (1.00-1.89) |
| Baseline Pulmonary Fibrosis | 6.53 (3.09-13.47) | 3.35 (1.38-8.13) |
| Baseline Ulceration | 4.06 (2.72-5.95) | 2.02 (1.28-3.19) |
| CVA (cerebrovascular accident) CKD3B (Chronic Kidney Disease Stage 3B), ESRD (End Stage Renal Disease), MI (Myocardial Infarction), PAD (Peripheral Arterial Disease), DM (Diabetes Mellitus)  *Imputed variable (smoking 1.4% missing, ethnicity 54.3% missing)  Mortality outcome = death ever during study period | | |

Supplementary Table S5 Extended Cox Proportional Hazards Model Showing Measured Association Between any Damage (eSDI>0) and Mortality

| **Variable** | **HR (95% CI)** | **P value** |
| --- | --- | --- |
| Damage (SDI >0) | 11.77(8.22-16.84) | <0.001 |
| Age at index | 1.05(1.04-1.06) | <0.001 |
| Ever smoker | 1.44(1.17-1.78) | <0.001 |
| Other ethnic group | 1.97(1.18-3.28) | <0.001 |
| Baseline MI | 1.76(1.13-2.74) | 0.01 |
| Baseline ESRD | 1.46(0.47-4.53) | 0.51 |
| Baseline Angina | 1.06(0.75-1.49) | 0.76 |
| Baseline CVA | 1.04(0.68-1.6) | 0.86 |
| Baseline DM | 1.22(0.85-1.75) | 0.29 |
| Baseline Heart failure | 1.36(0.81-2.29) | 0.25 |
| Baseline Malignancy | 1.44(1.08-1.91) | 0.01 |
| Baseline CKD3B | 1.8(1.15-2.82) | <0.001 |
| Baseline PAD | 1.4(0.94-2.08) | 0.10 |
| Baseline Pulmonary Fibrosis | 1.47(0.77-2.82) | 0.24 |
| Baseline Ulceration | 1.52(1.04-2.22) | 0.03 |
| CVA (cerebrovascular accident) CKD3B (Chronic Kidney Disease Stage 3B), ESRD (End Stage Renal Disease), MI (Myocardial Infarction), PAD (Peripheral Arterial Disease), DM (Diabetes Mellitus) | | |
|  | | |

Supplementary Table S6 Extended Cox Proportional Hazards Model Showing Measured Association Between any Cumulative Damage and Mortality

| **Variable** | **HR (95% CI)** | **P value** |
| --- | --- | --- |
| Age at index | 1.06(1.05-1.07) | <0.001 |
| Ever smoking | 1.55(1.27-1.88) | <0.001 |
| Non-White Ethnicity | 1.68(1.01-2.79) | 0.05 |
| Baseline MI | 1.33(0.88-2.01) | 0.17 |
| Baseline ESRF | 2.56(0.97-6.73) | 0.06 |
| Baseline Angina | 1.07(0.78-1.46) | 0.68 |
| Baseline CVA | 1.11(0.74-1.67) | 0.61 |
| Baseline DM | 1.1(0.77-1.56) | 0.60 |
| Baseline Heart failure | 1.75(1.06-2.9) | 0.03 |
| Baseline Malignancy | 1.3(1.01-1.69) | 0.05 |
| Baseline CKD3B | 1.75(1.16-2.66) | 0.01 |
| Baseline PAD | 1.35(0.92-1.98) | 0.12 |
| Baseline Ulceration | 1.56(1.11-2.2) | 0.01 |
| Cumulative damage (0-5 years) | 1.76(1.59-1.94) | <0.001 |
| Cumulative damage (5-10 years) | 1.7(1.56-1.85) | <0.001 |
| Cumulative damage (10-15 years) | 1.55(1.39-1.73) | <0.001 |
| Cumulative damage (15-20 years) | 1.58(1.34-1.86) | <0.001 |
| Cumulative damage (20-25 years) | 1.29(0.95-1.76) | 0.10 |
| Cumulative damage (25-30 years) | 2.42(0.48-12.14) | 0.28 |
| CVA (cerebrovascular accident) CKD3B (Chronic Kidney Disease Stage 3B), ESRD (End Stage Renal Disease), MI (Myocardial Infarction), PAD (Peripheral Arterial Disease), DM (Diabetes Mellitus) | | |
